# Comparative outcomes in patients receiving pirfenidone or nintedanib for idiopathic pulmonary fibrosis

**DOI:** 10.1186/s12931-021-01714-y

**Published:** 2021-05-04

**Authors:** Manon Belhassen, Faustine Dalon, Maëva Nolin, Eric Van Ganse

**Affiliations:** 1 PELyon, PharmacoEpidemiology Lyon, 210 avenue Jean Jaurès, 69007 Lyon, France; 2 Hospices Civils de Lyon, Croix-Rousse University Hospital, Department of Respiratory Medicine, 103 Grande Rue de la Croix-Rousse, 69004 Lyon, France; 3 RESearch on HealthcAre PErformance (RESHAPE), Claude Bernard Lyon 1 University, 8 avenue Rockefeller, 69003 Lyon, France

**Keywords:** Idiopathic pulmonary fibrosis, Antifibrotics, Mortality, Acute hospitalisations

## Abstract

**Background:**

Real-world data regarding outcomes of idiopathic pulmonary fibrosis (IPF) are scarce, outside of registries. In France, pirfenidone and nintedanib are only reimbursed for documented IPF, with similar reimbursement criteria with respect to disease characteristics, prescription through a dedicated form, and IPF diagnosis established in multidisciplinary discussion.

**Research question:**

The data of the comprehensive French National Health System were used to evaluate outcomes in patients newly treated with pirfenidone or nintedanib in 2015–2016.

**Study design and methods:**

Patients aged < 50 years or who had pulmonary fibrosis secondary to an identified cause were excluded. All-cause mortality, acute respiratory-related hospitalisations and treatment discontinuations up to 31 December 2017 were compared using a Cox proportional hazards model adjusted for age, sex, year of treatment initiation, time to treatment initiation and proxies of disease severity identified during a pre-treatment period.

**Results:**

During the study period, a treatment with pirfenidone or nintedanib was newly initiated in 804 and 509 patients, respectively. No difference was found between groups for age, sex, time to treatment initiation, Charlson comorbidity score, and number of hospitalisations or medical contacts prior to treatment initiation. As compared to pirfenidone, nintedanib was associated with a greater risk of all-cause mortality (hazard ratio [HR], 1.8; 95% confidence interval [CI] 1.3–2.6), a greater risk of acute respiratory-related hospitalisations (HR 1.3; 95% CI 1.0–1.7) and a lower risk of treatment discontinuation at 12 months (HR 0.7; 95% CI 0.6–0.9).

**Interpretation:**

This observational study identified potential differences in outcome under newly prescribed antifibrotic drugs, deserving further explorations.

**Supplementary Information:**

The online version contains supplementary material available at 10.1186/s12931-021-01714-y.

## Background

Idiopathic pulmonary fibrosis (IPF) is a chronic, relentlessly progressive and ultimately fatal lung disease of unknown aetiology [1]. The classic clinical phenotype of IPF is one of slowly progressive decline in lung function and worsening dyspnoea leading to death within 2–5 years of diagnosis if untreated, sometimes with interspersed episodes of acute worsening [2].

Pirfenidone was marketed in France in October 2012. The CAPACITY and ASCEND trials demonstrated a reduction in the decline in forced vital capacity (FVC) [3, 4], a reduction in the proportion of patients who had an absolute decline of 10% or more in predicted FVC or died [4], and an improvement in progression-free survival in pirfenidone-treated patients with IPF [3]. In addition, pirfenidone was associated with a reduction in the relative risk of overall mortality at week 52 in a pooled analysis of the CAPACITY and ASCEND trials (hazard ratio [HR] 0.52; 95% confidence interval [CI] 0.31–0.87; p = 0.01), and in a meta-analysis also including the Shionogi phase 2 and phase 3 Japanese trials [5]. Post-hoc analyses also suggested that pirfenidone treatment reduces the rate of non-elective hospitalisation in patients with IPF [6].

Nintedanib was marketed in France in April 2015, after a short period under Temporary Use Authorization (TUA). The two phase 3 INPULSIS trials showed that nintedanib significantly decreases FVC decline compared to placebo, but provided inconsistent results with regard to time to first acute exacerbation [7]. Moreover, in a pooled analysis of the phase 2 TOMORROW trial and the two phase 3 INPULSIS trials, the HR for time to first acute exacerbation was 0.53 (95% CI 0.34–0.83; p = 0.0047), and the HRs for time to all-cause and on-treatment mortality were 0.70 (95% CI 0.46–1.08; p = 0.0954) and 0.57 (95% CI 0.34–0.97; p = 0.0274), respectively, in favour of nintedanib [8].

Although all-cause mortality is arguably the most clinically relevant endpoint in IPF [9], conducting a trial with appropriate power and follow-up to demonstrate a survival benefit is difficult, if at all possible [10–12], particularly in the era of antifibrotic therapy wherein a placebo-controlled trial would be seen as unethical. In addition to pooled analysis of large trials, data from registries in Australia and Europe have showed better survival in patients with IPF receiving antifibrotic drugs [13, 14]. However, real-world survival data from large unselected European patient populations and comparison between the two drugs are still scarce. Here, we compared all-cause mortality and acute respiratory-related hospitalisation rates in patients with IPF receiving pirfenidone or nintedanib, using data from the French National Health System (NHS).

## Methods

### Data source

This historical, population-based cohort study used digital data from the French NHS, or Système National des Données de Santé [SNDS], which covers 98.8% of the population living in France. This unique real-world dataset of French healthcare utilisation is one of the largest data repositories worldwide. It contains comprehensive, anonymous, individual information on sociodemographic characteristics, date of death, out-of-hospital reimbursed healthcare expenditures (from both public and private healthcare), and hospital discharge summaries with International Classification of Diseases (ICD)-10 codes [15]. In addition, the SNDS contains direct information on medical diagnoses for patients who have full coverage for all medical expenses by the NHS (‘chronic disease status’), including most patients diagnosed with IPF in France.

### Drug use and reimbursement

Pirfenidone and nintedanib are currently the only approved treatments for IPF in France. Following international [16] and French national [17] guidelines, both drugs have the same indication and reimbursement modalities in France, i.e. diagnosis of IPF confirmed in a multidisciplinary setting, FVC ≥ 50% of predicted value and diffusing capacity of the lung for carbon monoxide ≥ 30% of predicted value [18, 19]. Prescription is restricted to pulmonologists using a dedicated form, to certify that patients receiving pirfenidone or nintedanib actually have IPF.

### Study population

The study population consisted of patients with IPF who were newly treated with pirfenidone or nintedanib between 1 January 2015 and 31 December 2016 (inclusion period). To ensure analytical data exhaustivity, only patients continuously covered by the French NHS during the study period (between 1 January 2010 and 31 December 2017) were included. Patients younger than 50 were excluded, as were patients with pulmonary fibrosis other than IPF during the study period (see Additional file 1: Table S1) and patients who had previously received a lung transplant. To select patients who were newly treated, those who had received pirfenidone or nintedanib in the five years before the inclusion period were excluded. As data from SNDS are made available with some delay, 2017 was the last year available at the time of the analyses and follow-up was censored as of 31 December 2017.

### Study outcomes

The outcomes of interest were all-cause mortality, acute respiratory-related hospitalisation and treatment discontinuation at 12 months. Acute respiratory-related hospitalisations were defined as hospitalisations due to acute respiratory events, either triggered or idiopathic, identified using the main diagnoses of respiratory-related hospitalisations (see Additional file 1: Table S2).

### Statistical analysis

Time to first occurrence of all-cause mortality or acute respiratory-related hospitalisation was estimated using cumulative incidence functions (using the day of treatment initiation as baseline). For each patient, differences in follow-up were accounted for through non-informative censoring at the end of follow-up. The competing risk of mortality was accounted for through informative censoring at death when mortality was > 10%.

Treatment initiation date was defined as the first day when the drug was dispensed by a pharmacy without the use of any antifibrotic medication in the five previous years. Patients were followed-up from the treatment initiation until their last health record, lung transplantation, death, or the end of the study period (31 December 2017), whichever occurred first.

The drug-exposure period was defined as the time between the date of treatment initiation and the occurrence of one of the following events, whichever came first: a switch to another antifibrotic treatment (from nintedanib to pirfenidone or vice versa), treatment discontinuation (*i.e.* failure to refill a prescription within 30 days of the end of supply), or the end of the follow-up period. Patients newly treated with nintedanib and those newly treated with pirfenidone were compared over the exposure period using a Cox proportional hazards model adjusted for confounding factors (time-to-event analyses).

The following confounding factors were taken into account in comparisons between groups: age at treatment initiation, sex, year of treatment initiation (2015 or 2016), time in months between the diagnosis of pulmonary fibrosis and the date of treatment initiation, and proxies of disease severity identified during the year prior to treatment initiation: number of acute respiratory-related hospitalisations, number of outpatient visits to a hospital physician, Charlson comorbidity index and use of oxygen therapy. The Charlson comorbidity index has been specifically developed and validated by the French NHS for use in SNDS studies [20].

All statistical analyses were performed using SAS (SAS Institute, North Carolina, US), version 9.4.

### Ethics

This study was approved by the French Institute for Health Data (approval no. 57932 from 12 July 2018). It was conducted with anonymised data, as requested by the National Informatics and Liberty Commission [CNIL], approval no. 918255, from 8 August 2018.

## Results

### Study population

**Fig. 1:**
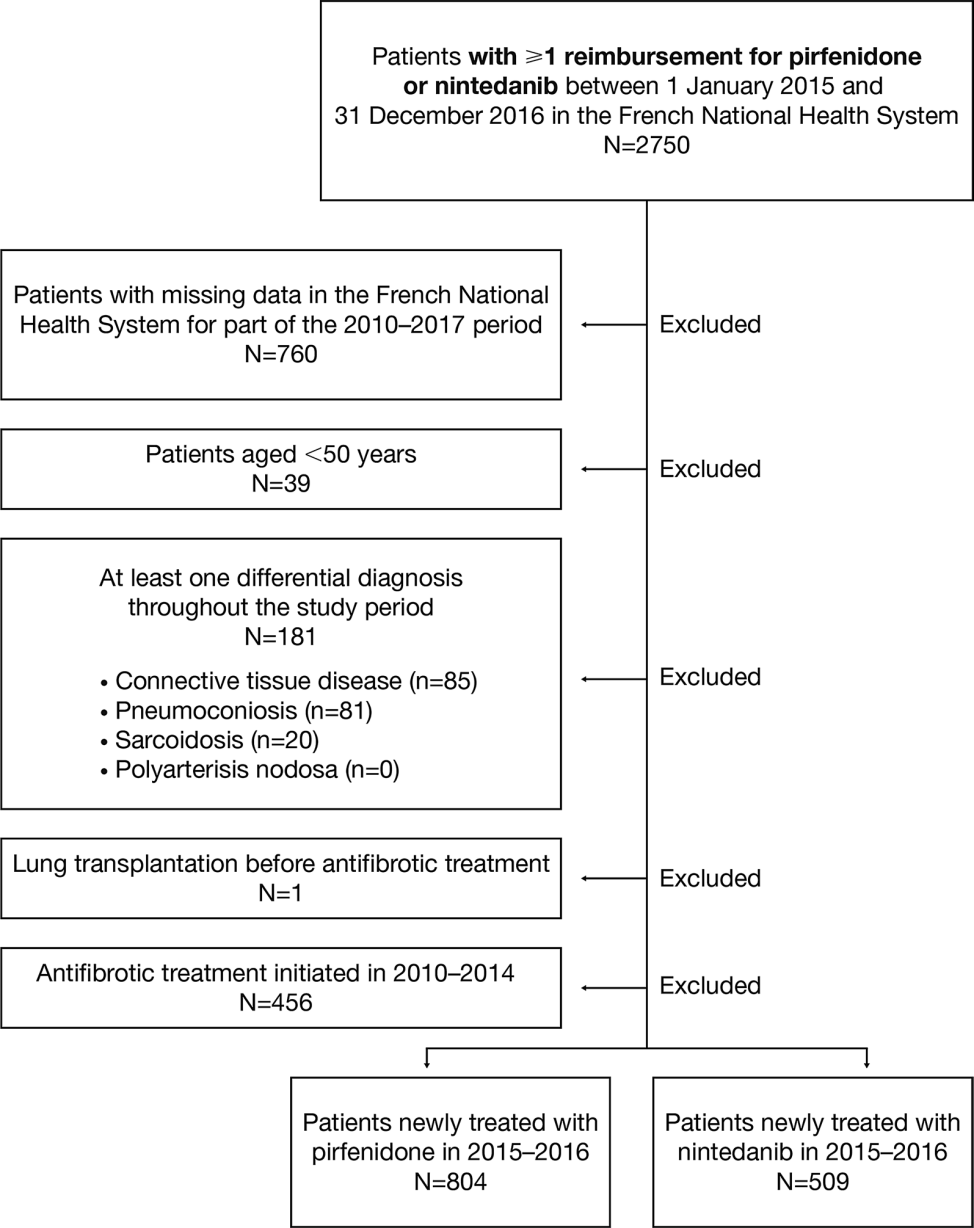
Patient flowchart

The flowchart of patients’ selection is shown in Fig. 1. Of 2750 eligible patients, 804 and 509 patients were newly treated with pirfenidone or nintedanib, respectively, and were included in the analysis. Two patients (0.4%) receiving nintedanib in the context of Temporary Use Authorization (TUA), i.e. between January and April 2015, were included in the study population. In both groups, about three-quarters of the patients were males, with a mean age of approximately 73 years (Table 1). Treatment was initiated more frequently with pirfenidone than with nintedanib in 2015, while the reverse was true in 2016. About 40% of patients had an acute respiratory-related hospitalisation during the year prior to treatment initiation (in both groups). More than half of patients had a Charlson comorbidity score of 3 or 4. No significant difference was found between groups for age, sex, time from IPF diagnosis to antifibrotic treatment initiation, Charlson comorbidity score, use of supplemental oxygen, number of acute respiratory-related hospitalisations and number of outpatient visits to a hospital physician during the year prior to treatment initiation.

**Table 1:** Baseline characteristics of patients newly treated with an antifibrotic drug

Characteristic	Patients newly treated with pirfenidone (N = 804)	Patients newly treated with nintedanib (N = 509)	p-value
Age at treatment initiation, years			
Mean (SD)	73.2 (8.1)	72.8 (8.1)	0.49
Median (IQR)	74.0 (67.0–79.0)	73.0 (68.0–79.0)	
Minimum–maximum	50.0–92.0	51.0–97.0	
Sex, n (%)			
Male	618 (76.9)	391 (76.8)	0.98
Female	186 (23.1)	118 (23.2)	
Year of treatment initiation, n (%)			
2015	513 (63.8)	86 (16.9)	< 0.01
2016	291 (36.2)	423 (83.1)	
Time from IPF diagnosis to treatment initiation, months			
Mean (SD)	9.0 (15.0)	10.6 (18.0)	0.26
Median (IQR)	2.3 (0.0–10.2)	2.3 (0.0–11.2)	
Minimum–maximum	0.0–96.8	0.0–100.6	
Number of acute respiratory-related hospital admissions during the year prior to treatment initiation, n (%)			
0	476 (59.2)	314 (61.7)	0.58
1	247 (30.7)	151 (29.7)	
2 or more	81 (10.1)	44 (8.6)	
Number of outpatient visits to a hospital physician during the year prior to treatment initiation, n (%)			
0	83 (10.3)	48 (9.4)	0.74
1 or 2	252 (31.3)	170 (33.4)	
3 or 4	216 (26.9)	142 (27.9)	
5 or more	253 (31.5)	149 (29.3)	
Charlson comorbidity score, n (%)			
1–2	122 (15.2)	85 (16.7)	0.48
3–4	436 (54.2)	283 (55.6)	
5 or more	246 (30.6)	141 (27.7)	
Use of supplemental oxygen at baseline, n (%)			
No	611 (76.0)	373 (73.3)	0.27
Yes	193 (24.0)	136 (26.7)	

*IPF* idiopathic pulmonary fibrosis, *IQR* interquartile range, *SD* standard deviation

### All-cause mortality

**Fig. 2:**
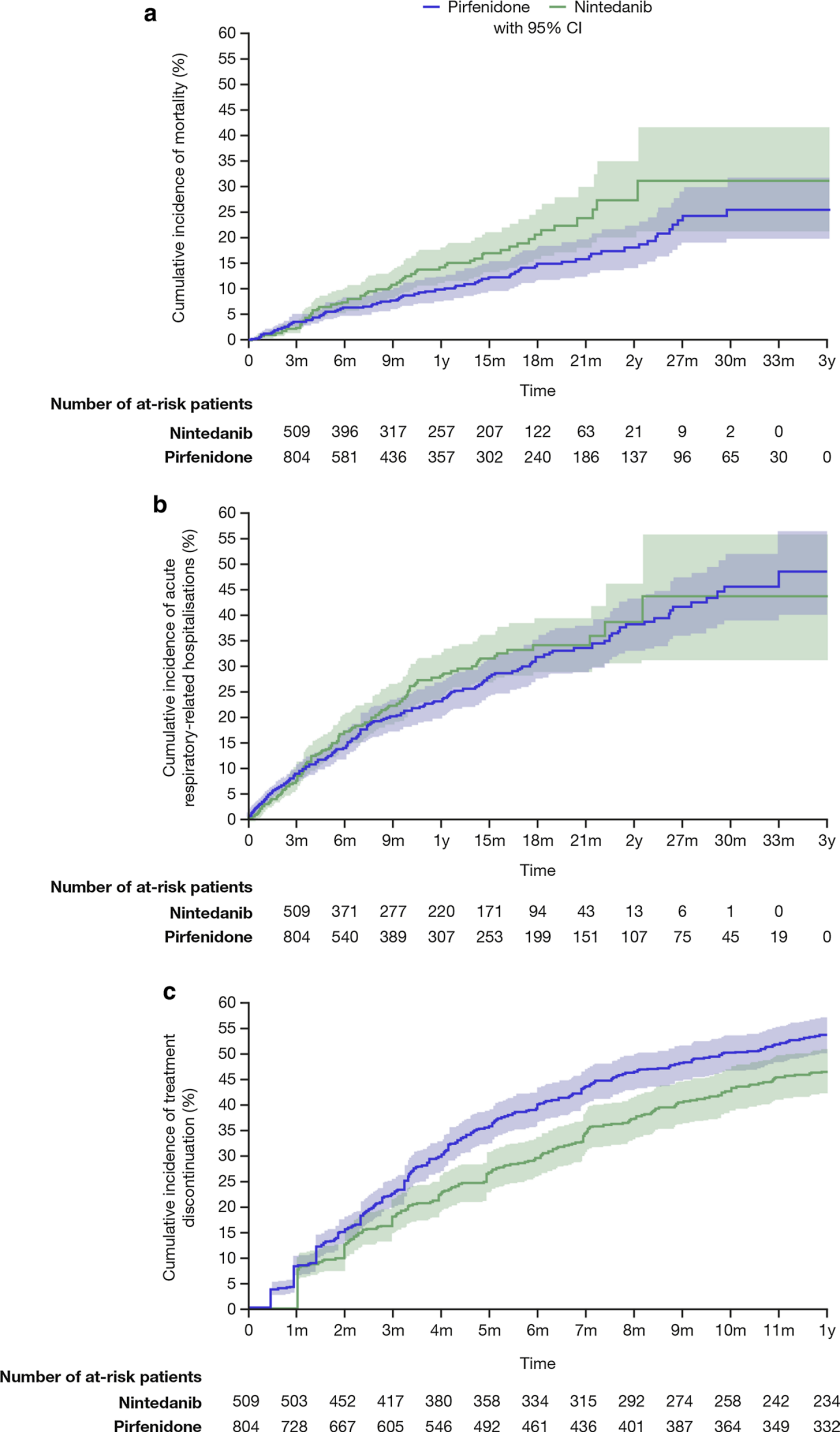
Comparison of outcomes in patients newly treated with pirfenidone or nintedanib. **a** Comparison of overall mortality in patients newly treated with pirfenidone or nintedanib;^a^ **b** Comparison of acute respiratory-related hospital admissions during the drug-exposure period in patients newly treated with pirfenidone or nintedanib;^b^ **c** Comparative incidence of discontinuation of pirfenidone or nintedanib treatment.^b^ *CI* confidence interval, *m* month, *y* year. ^a^Cumulative mortality was estimated using a cumulative incidence function. ^b^Cumulative incidence was estimated using a cumulative incidence function. The coloured areas represent the 95% CI

The unadjusted mortality rate was 10.9 per 100 person-years (95% CI 8.8–13.5) and 15.4 per 100 person-years (95% CI 12.1–19.4) in patients newly treated with pirfenidone and nintedanib, respectively. The cumulative mortality at three years was 25.5% (95% CI 19.6–31.7) and 31.1% (95% CI 21.2–41.6) in patients newly treated with pirfenidone and nintedanib, respectively (Table 2; Fig. 2a).

**Table 2:** Unadjusted incidence rates and cumulative incidence of events in patients newly treated with antifibrotic drugs

	Unadjusted incidence rate [95% CI] (per 100 person-years)	One-year cumulative incidence of event, % [95% CI]	Three-year cumulative incidence of event, % [95% CI]
*All-cause mortality*			
Patients initiating pirfenidone	10.91 [8.81–13.51]	9.79 [7.41–12.56]	25.46 [19.60–31.71]
Patients initiating nintedanib	15.36 [12.13–19.44]	14.25 [10.81–18.14]	31.11 [21.16–41.56]
*Acute respiratory-related hospital admissions*			
Patients initiating pirfenidone	26.99 [23.34–31.21]	22.85 [19.35–26.52]	48.26 [39.76–56.24]
Patients initiating nintedanib	30.70 [25.69–36.68]	27.46 [22.95–32.14]	43.62 [30.75–55.79]
*Treatment discontinuation*			
Patients initiating pirfenidone	82.88 [75.27–91.26]	53.75 [50.13–57.22]	–
Patients initiating nintedanib	64.82 [56.85–73.91]	46.53 [41.96–50.97]	–

*CI* confidence interval

The unadjusted HR for all-cause mortality in nintedanib- *versus* pirfenidone-treated patients was 1.4 (95% CI 1.0–2.0). After adjustment for confounding factors, the HR for all-cause mortality in nintedanib- versus pirfenidone-treated patients was 1.8 (95% CI 1.3–2.6). Variables associated with increased mortality were age, year of treatment initiation, two or more acute respiratory-related hospital admissions during the year prior to treatment initiation, Charlson score of 5 or greater, and use of supplemental oxygen (Table 3).

**Table 3:** Adjusted comparison of all-cause mortality in patients newly treated with antifibrotic drugs (multivariate analyses)

	HR	95% CI	p-Value
Treatment			
Pirfenidone	1.00	–	–
Nintedanib	1.80	1.25–2.60	< 0.01
Age at treatment initiation	1.03	1.01–1.06	0.02
Sex			
Male	1.00	–	–
Female	0.65	0.42–1.02	0.06
Year of antifibrotic treatment initiation			
2015	1.00	–	–
2016	0.62	0.42–0.91	0.01
Time from IPF diagnosis to treatment initiation	1.00	0.99–1.01	0.51
Number of acute respiratory-related hospital admissions during the year prior to treatment initiation			
0	1.00	–	–
1	1.29	0.89–1.85	0.18
2 or more	1.70	1.09–2.64	0.02
Number of outpatient visits to a hospital physician during the year prior to treatment initiation			
0	1.00	–	–
1 or 2	1.22	0.59–2.50	0.59
3 or 4	1.19	0.56–2.53	0.65
5 or more	1.85	0.90–3.79	0.09
Charlson comorbidity score			
1–2	1.00	–	–
3–4	1.57	0.79–3.13	0.19
5 or more	2.48	1.19–5.16	0.02
Use of supplemental oxygen at baseline			
Yes	3.27	2.34–4.58	< 0.01

*CI* confidence interval, *HR* hazard ratio, *IPF* idiopathic pulmonary fibrosis

For the sub-group of patients initiating the treatment in 2016, the model (adjusted for the same confounding factors) showed an HR = 1.38 (95% CI 0.83–2.30) (see Additional file 1: Table S3 and Figure S1).

### Acute respiratory-related hospital admissions

The unadjusted incidence rate of acute respiratory-related hospital admission was 27.0 per 100 person-years (95% CI 23.3–31.2) and 30.7 per 100 person-years (95% CI 25.7–36.7), in patients newly treated with pirfenidone and nintedanib, respectively. The cumulative incidence of the event at three years was 48.3% (95% CI 39.8–56.2) and 43.6% (95% CI 30.8–55.8), in patients newly treated with pirfenidone and nintedanib, respectively (Table 2; Fig. 2b).

The unadjusted HR for acute respiratory-related hospital admission in nintedanib- *versus* pirfenidone-treated patients was 1.1 (95% CI 0.9–1.4). After adjustment for all confounding factors, the HR for acute respiratory-related hospital admission in nintedanib- *versus* pirfenidone-treated patients was 1.3 (95% CI 1.0–1.7).Variables associated with an increased risk of acute respiratory-related hospital admission were year of treatment initiation, time from IPF diagnosis to treatment initiation, number of acute respiratory-related hospital admissions during the year prior to treatment initiation and use of supplemental oxygen (Table 4).

**Table 4:** Adjusted comparison of acute respiratory-related hospitalisation in patients newly treated with antifibrotic drugs (multivariate analyses)

	HR	95% CI	p-value
Treatment			
Pirfenidone	1.00	–	–
Nintedanib	1.32	1.01–1.73	0.04
Age at treatment initiation	0.99	0.98–1.01	0.41
Sex			
Male	1.00	–	–
Female	0.98	0.74–1.30	0.90
Year of antifibrotic treatment initiation			
2015	1.00	–	–
2016	0.70	0.53–0.91	0.01
Time from IPF diagnosis to treatment initiation	1.01	1.00–1.01	0.02
Number of acute respiratory-related hospital admissions during the year prior to treatment initiation			
0	1.00	–	–
1	1.42	1.10–1.84	0.01
2 or more	2.27	1.64–3.15	< 0.01
Number of outpatient visits to a hospital physician during the year prior to treatment initiation			
0	1.00	–	–
1 or 2	1.08	0.68–1.72	0.75
3 or 4	0.99	0.61–1.60	0.97
5 or more	1.11	0.69–1.78	0.66
Charlson comorbidity score			
1–2	1.00	–	–
3–4	1.33	0.91–1.94	0.14
5 or more	1.35	0.87–2.09	0.18
Use of supplemental oxygen at baseline			
Yes	2.02	1.57–2.60	< 0.01

*CI* confidence interval, *HR* hazard ratio, *IPF* idiopathic pulmonary fibrosis

### Discontinuation of antifibrotic drug

Pirfenidone treatment was discontinued within 12 months of initiation in 51.5% of newly treated patients after a mean of 126.4 (91.4) days. The unadjusted incidence rate of discontinuation was 82.9 per 100 person-years (95% CI 75.3–91.3) and the cumulative incidence of discontinuation at one year was 53.8% (95% CI 50.1–57.2). Nintedanib treatment was discontinued within 12 months of initiation in 43.8% of patients, after a mean of 142.5 (95.7) days. The unadjusted incidence rate of discontinuation was 64.8 per 100 person-years (95% CI 56.9–73.9) and the cumulative incidence of discontinuation at one year was 46.5% (95% CI 42.0–51.0) (Table 2; Fig. 2c).

The unadjusted HR for discontinuation at 12 months in nintedanib- versus pirfenidone-treated patients was 0.8 (95% CI 0.7–0.9). After adjustment for all confounding factors, the HR for discontinuation at 12 months in nintedanib- versus pirfenidone-treated patients was 0.7 (95% CI 0.6–0.9). Variables associated with an increased risk of treatment discontinuation were age, sex and use of supplemental oxygen (Table 5).

**Table 5:** Adjusted comparison of treatment discontinuation at 12 months in patients newly treated with antifibrotic drugs

	HR	95% CI	p-value
Treatment			
Pirfenidone	1.00	–	–
Nintedanib	0.72	0.60–0.86	< 0.01
Age at treatment initiation	1.03	1.01–1.04	< 0.01
Sex			
Male	1.00	–	–
Female	1.37	1.15–1.63	< 0.01
Year of antifibrotic treatment initiation			
2015	1.00	–	–
2016	1.15	0.96–1.37	0.12
Time from IPF diagnosis to treatment initiation			
≤ 1 month	1.00	–	–
1–12 months	0.86	0.70–1.06	0.15
> 12 months	1.10	0.90–1.34	0.35
Number of acute respiratory-related hospital admissions during the year prior to treatment initiation			
0	1.00	–	–
1	0.83	0.68–1.00	0.06
2 or more	0.80	0.58–1.11	0.18
Number of outpatient visits to a hospital physician during the year prior to treatment initiation			
0	1.00	–	–
1 or 2	0.82	0.62–1.07	0.14
3 or 4	0.99	0.75–1.30	0.94
5 or more	0.91	0.69–1.20	0.52
Charlson comorbidity score			
1–2	1.00	–	–
3–4	1.03	0.78–1.36	0.83
5 or more	0.93	0.67–1.29	0.67
Use of supplemental oxygen at baseline			
Yes	1.21	1.00–1.45	0.05

*CI* confidence interval, *HR* hazard ratio, *IPF* idiopathic pulmonary fibrosis

## Discussion

In this historical cohort study, clinically relevant outcomes were compared between patients with IPF newly treated with pirfenidone (n = 804) and nintedanib (n = 509), using the French national claims dataset. After adjustment on confounding factors, at one year, nintedanib use was associated with a higher all-cause mortality (HR, 1.8; 95% CI 1.3–2.6), a higher risk of acute respiratory-related hospital admission (HR, 1.3; 95% CI 1.0–1.7) and a lower risk of treatment discontinuation (HR, 0.7; 95% CI 0.6–0.9) compared to pirfenidone use.

Comparisons were adjusted for several variables known to be associated with mortality in patients with IPF. Consistent with previous studies, age [2, 21, 22], Charlson comorbidity index [23], use of supplemental oxygen [24] and hospital admissions in the year prior to treatment initiation [25] were associated with increased risks of all-cause mortality. Number of hospital admissions in the year prior to treatment initiation, time between IPF diagnosis and treatment initiation, and use of supplemental oxygen were also associated with acute respiratory-related hospitalisations in the study period, similar to previous studies [2, 22, 24, 25]. Adjustment on variables made it possible to assess the influence of each confounding factors on outcomes. The use of propensity score method was not necessary in this study as there were enough events to be able to include all the variables in multivariable analyses.

As this study was based on claims data, lung-function parameters and their changes with time, which are predictors of outcome in patients with IPF, were not available. However, both drugs have the same indication, prescription and reimbursement modalities in France, and both study groups had similar characteristics including time to treatment initiation. It is therefore unlikely that the observed differences in outcome could be explained by differences in disease severity at the time of antifibrotic treatment initiation. Furthermore, as the study was focused on the initial period of use of IPF-therapy (2015–2016), when physicians were more experienced with the use of pirfenidone, we decided to perform a distinct analysis for patients initiating a treatment in 2016. Indeed, in 2016, one can reasonably assume that clinicians had acquired an appropriate and comparable experience with the two products, which targeted similar IPF populations and which were both initiated at the same moment of disease progression, with similar functional status of the patients in the two groups. This analysis suggested a numerically higher risk of mortality with nintedanib treatment for the patients included in 2016. As prescription of antifibrotic medications is strictly controlled in France by the use of a dedicated prescription form for the sole indication of IPF, restriction of prescription to pulmonologists and requirement of diagnosis confirmation following multidisciplinary discussion, we are confident that the study population consisted of patients with a ‘true’ diagnosis of IPF. Moreover, the off-label prescription of antifibrotic drugs is expected to be very rare. Indeed, in the large international PASSPORT registry, only three of 1009 patients enrolled (and none for the subgroup from France) had a diagnosis other than IPF, despite the fact that this registry allowed the inclusion of subjects with other indications [26]. Therefore, although the diagnosis was not confirmed individually for each patient, we are confident that prescription of antifibrotic medications was limited to patients with IPF. An algorithm based on age and ICD-10 codes was nevertheless used to exclude subjects who may have had a differential diagnosis, especially connective tissue disease, sarcoidosis and pneumoconiosis. The median age in our population (73 years) and the large male-to-female predominance are consistent with a diagnosis of IPF in the vast majority of our patients.

To date, no head-to-head randomised controlled trials have been conducted to compare pirfenidone and nintedanib. Indirect comparisons (network meta-analyses) have compared the two treatments, generally showing no clear difference in outcome between the two drugs, although with some discrepancy [27–29] and methodological limitations. The network meta-analysis performed by Fleetwood et al. [29] was however the only one that could assess outcomes at 52 weeks after initiation in all treatment groups, suggesting a lower, but not significant all-cause mortality with pirfenidone compared to nintedanib (HR = 0.74; 95% CI 0.27–1.95). In a previous analysis based on insurance claims data in the US, Dempsey et al. [30] found (in a study population of comparable size to ours) a decreased risk of all-cause mortality in patients receiving an antifibrotic drug, but no significant difference in all-cause mortality between drugs (nintedanib versus pirfenidone, HR for overall mortality, 1.14; 95% CI 0.79–1.65; p = 0.471). The reasons for the discrepancy between the study by Dempsey and ours can only be speculated upon. Dempsey et al. [30] used data from OptumLabs Data Warehouse, which includes data from both Medicare and commercially insured subjects, *e.g.* presumably a group of patients with a smaller fraction of low-income or unemployed individuals, as compared to the French NHS data that cover the entire population. In addition, both drugs became available at the same time in the US, while in France nintedanib was reimbursed 30 months later than pirfenidone, potentially influencing physician choice of the medication. Although both pirfenidone and nintedanib were approved and reimbursed in the US for the same indication (i.e. treatment of IPF, irrespective of disease severity), co-payments may vary and influence treatment initiation, continuation or choice of medication. Of note, in the Dempsey study, the discontinuation rate was high (45.1%) but no information was given about between-drug differences in the rate of discontinuation.

The choice of initiating pirfenidone or nintedanib could, in theory, be related to patient characteristics, disease history, comorbidities, concomitant medications, physician experience and patient choice based on possible adverse events. The earlier availability of pirfenidone compared to nintedanib in France could have impacted how the drugs were prescribed during the study period. To limit the impact of known or unknown confounders, our study included only first-time users, and the models were adjusted by date of first prescription and time between the date of IPF diagnosis and treatment initiation. Of note, in our study, two patients received nintedanib in the context of a TUA (i.e. with potentially a more severe disease), but they made only 0.4% of the nintedanib sample, which therefore could not impact the results.

Overall, more than 15% of the patients stopped therapy early after treatment initiation (within 3 months) and 50% of patients stopped their treatment at one year. Comparative analysis of treatment discontinuation showed that female patients were more likely to interrupt therapy, in agreement with a previous study conducted in patients with chronic obstructive pulmonary disease [31]. The use of supplemental oxygen, a proxy of disease severity, was associated with a greater risk of treatment discontinuation, in contrast to other studies showing fewer interruptions with increasing disease severity [31]. Pirfenidone treatment was associated with a higher risk of drug discontinuation at 12 months than nintedanib. Treatment discontinuation could have been due to tolerability issues, which tend to occur within three months of treatment initiation [32], or to a switch from pirfenidone to nintedanib. However, results from this study represent on-treatment cumulative incidence up to one year and therefore are unlikely to be influenced by a switch in medication.

This study had some limitations mainly related to its observational design and data source. The ICD-10 code used to confirm the presence of pulmonary fibrosis in the study population (J84.1) is not specific to IPF, and ICD codes and age were mostly used to exclude other forms of fibrotic interstitial lung diseases among patients initiating an antifibrotic drug. Although imperfect, such an approach has already been used in the French healthcare dataset in a study that further validated the definition algorithm for acute respiratory-related hospitalisations [33]. Risk factors for outcomes were identified from proxies based on hospital admissions, visits, concomitant medications and hospital discharge codes; however, data on lung function and body mass index, both major determinants of IPF prognosis, were not available, as is often the case for claims data. While the effects of confounding factors were taken into account as thoroughly as possible, residual confounding effects may still be present due to the study design. Nonetheless, the consistency of our findings with regard to all-cause mortality and acute respiratory-related hospitalisations, along with identifying factors known to impact these outcomes, such as disease severity or prior hospital admissions, supports the robustness of our findings.

Among observational studies of antifibrotics, this study stands out because it was population-based, conducted with a dataset recording the health care consumption of almost all French citizens (i.e. a population of 60+ million inhabitants, without distinction of age, gender, ethnicity, residency, incomes, psychosocial status, etc., i.e. from a large country with universal healthcare coverage and a single unified healthcare system), and because it records individual and comprehensive data for the population since 2006.

## Conclusion

In summary, this comparative analysis of outcomes following initiation of antifibrotic therapy for IPF suggests a lower all-cause mortality under pirfenidone than under nintedanib. This must obviously be confirmed by additional studies on larger samples and with other databases, as we cannot exclude residual confounding factors.

## Supplementary Information

**Additional file 1: Table S1.** ICD-10 codes for exclusion criteria. **Table S2.** ICD-10 codes for acute respiratory-related hospitalisations (main diagnosis). **Table S3.** Unadjusted incidence rates and cumulative incidence of mortality in patients newly treated in 2016 with antifibrotic drugs. **Figure S1.** Comparison of overall mortality in patients newly treated in 2016 with pirfenidone or nintedanib.

## Data Availability

Due to NHS and SNDS rules, no data sharing is possible as access to data is restricted to habilitated and qualified researchers (Maeva Nolin is habilitated and qualified).

## Abbreviations

CI: Confidence interval
FVC: Forced vital capacity
HR: Hazard ratio
ICD: International classification of diseases
IPF: Idiopathic pulmonary fibrosis
NHS: National Health System
SNDS: Système National des Données de Santé – national system of health data
TUA: Temporary use authorization
