# Supplementary material for: Comparative outcomes in patients receiving pirfenidone or nintedanib for idiopathic pulmonary fibrosis
Source: Respir Res. 2021 May 4;22:135. doi: 10.1186/s12931-021-01714-y (PMC8094468; doi:10.1186/s12931-021-01714-y)
Supplement: Supplementary file 1 — Additional file 1: Table S1. ICD-10 codes for exclusion criteria. Table S2. ICD-10 codes for acute respiratory-related hospitalisations (main diagnosis). Table S3. Unadjusted incidence rates and cumulative incidence of mortality in patients newly treated in 2016 with antifibrotic drugs. Figure S1. Comparison of overall mortality in patients newly treated in 2016 with pirfenidone or nintedanib. [file 12931_2021_1714_MOESM1_ESM.docx]

**Additional tables**

**Additional table 1** ICD-10 codes for exclusion criteria

| **Diagnosis** | **Label** | **Hospitalisation** | **CD status** |
| --- | --- | --- | --- |
| Connective tissue diseases | Rheumatoid lung disease | J990 | – |
|  | Rheumatoid lung disease | M051 | – |
|  | Other necrotising vasculopathies | M31 | M31 |
|  | Systemic lupus erythematosus | M32 | M32 |
|  | Dermatopolymyositis | M33 | M33 |
|  | Systemic sclerosis | M34 | M34 |
|  | Other systemic involvement of connective tissue | M35 | M35 |
|  | Other localised connective tissue disorders | L94 | L94 |
| Pneumoconiosis | Coalworker pneumoconiosis | J60 | J60 |
|  | Pneumoconiosis due to asbestos and other mineral fibres | J61 | J61 |
|  | Pneumoconiosis due to dust containing silica | J62 | J62 |
|  | Pneumoconiosis due to other inorganic dust | J63 | J63 |
|  | Unspecified pneumoconiosis | J64 | J64 |
|  | Pneumoconiosis associated with tuberculosis | J65 | J65 |
|  | Airway disease due to specific organic dust | J66 | J66 |
|  | Hypersensitivity pneumonitis due to organic dust | J67 | J67 |
|  | Pleural plaque with presence of asbestos | J920 | – |
| Sarcoidosis | Sarcoidosis | D86 | D86 |
| Polyarteritis nodosa | Polyarteritis nodosa and related conditions | M30 | M30 |

CD: chronic disease; ICD-10: 10th revision of the International Statistical Classification of Diseases and Related Health Problems.

**Additional table 2** ICD-10 codes for acute respiratory-related hospitalisations (main diagnosis)

| **Diagnosis** | **Label** | **Hospitalisation** |
| --- | --- | --- |
| Legionnaires’ disease | Legionnaires’ disease | A481 |
| HIV disease resulting in infectious and parasitic diseases | HIV disease resulting in *Pneumocystis carinii* pneumonia | B206 |
| Cytomegaloviral pneumonitis | Cytomegaloviral pneumonitis | B250 |
| Pneumocystosis | Pneumocystosis | B59 |
| Influenza | Influenza due to identified zoonotic or pandemic influenza virus | J09 |
|  | Influenza due to identified seasonal influenza virus | J10 |
|  | Influenza, virus not identified | J11 |
| Pneumonia | Viral pneumonia, not classified elsewhere | J12 |
|  | Pneumonia due to *Streptococcus pneumoniae* | J13 |
|  | Pneumonia due to *Haemophilus influenzae* | J14 |
|  | Bacterial pneumonia not classified elsewhere | J15 |
|  | Pneumonia due to other infectious organisms not classified elsewhere | J16 |
|  | Pneumonia in diseases classified elsewhere | J17 |
|  | Pneumonia, organism unspecified | J18 |
| Bronchitis (not specified as acute or chronic) | Bronchitis not specified as acute or chronic | J40 |
| Bronchitis | Acute bronchitis | J20 |
| Acute bronchiolitis | Acute bronchiolitis | J21 |
| Unspecified acute lower respiratory infection | Unspecified acute lower respiratory infection | J22 |
| Other interstitial pulmonary diseases with fibrosis | Other interstitial pulmonary diseases with fibrosis | J841 |
| Pneumothorax | Pneumothorax | J93 |
| Acute respiratory failure | Acute respiratory failure | J960 |
| Chronic respiratory failure | Chronic respiratory failure | J961 |
| Unspecified respiratory failure | Respiratory failure, unspecified | J969 |

HIV: human immunodeficiency virus; ICD-10: 10th revision of the International Statistical Classification of Diseases and Related Health Problems.

**Additional table 3** Unadjusted incidence rates and cumulative incidence of mortality in patients newly treated in 2016 with antifibrotic drugs

|  | ***Pirfenidone N = 291*** | ***Nintedanib N = 423*** |
| --- | --- | --- |
| **Number of patients with event (%)** | | |
| Overall | 23 (7.9%) | 45 (10.6%) |
| **Duration between index date and event (in days)*** | | |
| Mean (Std) | 201.9 (189.6) | 267.2 (164.1) |
| Median (IQR) | 121.0 (45.0 - 322.0) | 241.0 (122.0 - 372.0) |
| Min - Max | 9.0 - 619.0 | 14.0 - 651.0 |
| **Total person-years of the at risk population** | 226.1 | 365.1 |
| **Unajusted incidence rate [95% CI] (per 100 person-years)** | 10.17 [6.76 - 15.30] | 12.32 [9.20 - 16.51] |
| **Cumulative incidence of event [95% CI]** | | |
| 1 year | 9.07% [5.46% - 13.80%] | 11.36% [7.97% - 15.41%] |
| 1.5 year | 13.70% [8.40% - 20.29%] | 19.27% [13.70% - 25.55%] |
| 2 years | 16.40% [9.48% - 24.97%] | 24.23% [15.66% - 33.84%] |

The mean age of patients included in 2016 was similar in both groups (74.0 years (SD=7.9) in the pirfenidone group versus 72.9 years (SD=8.1) in the nintedanib group), as well as the median age (75 years versus 74 years).

**Additional figure 1** Comparison of overall mortality in patients newly treated in 2016 with pirfenidone or nintedanib
